# Nanodosimetric Calculations of Radiation-Induced DNA Damage in a New Nucleus Geometrical Model Based on the Isochore Theory

**DOI:** 10.3390/ijms23073770

**Published:** 2022-03-29

**Authors:** Yann Thibaut, Nicolas Tang, Hoang Ngoc Tran, Aurélie Vaurijoux, Carmen Villagrasa, Sébastien Incerti, Yann Perrot

**Affiliations:** 1 Institut de Radioprotection et de Sûreté Nucléaire (IRSN), BP 17, 92262 Fontenay-aux-Roses, France; yann.thibaut@irsn.fr (Y.T.); aurelie.vaurijoux@irsn.fr (A.V.); carmen.villagrasa@irsn.fr (C.V.); 2 CHU Poitiers, Unité de Physique Médicale, Département de Radiothérapie, 86021 Poitiers, France; nicolas.tang78@gmail.com; 3 CNRS/IN2P3, CENBG, UMR 5797, Bordeaux University, 33170 Gradignan, France; tran@cenbg.in2p3.fr (H.N.T.); incerti@cenbg.in2p3.fr (S.I.)

**Keywords:** DNA damage simulation, Geant4-DNA, isochores

## Abstract

Double-strand breaks (DSBs) in nuclear DNA represents radiation-induced damage that has been identified as particularly deleterious. Calculating this damage using Monte Carlo track structure modeling could be a suitable indicator to better assess and anticipate the side-effects of radiation therapy. However, as already demonstrated in previous work, the geometrical description of the nucleus and the DNA content used in the simulation significantly influence damage calculations. Therefore, in order to obtain accurate results, this geometry must be as realistic as possible. In this study, a new geometrical model of an endothelial cell nucleus and DNA distribution according to the isochore theory are presented and used in a Monte Carlo simulation chain based on the Geant4-DNA toolkit. In this theory, heterochromatin and euchromatin compaction are distributed along the genome according to five different families (L1, L2, H1, H2, and H3). Each of these families is associated with a different hetero/euchromatin rate related to its compaction level. In order to compare the results with those obtained using a previous nuclear geometry, simulations were performed for protons with linear energy transfers (LETs) of 4.29 keV/µm, 19.51 keV/µm, and 43.25 keV/µm. The organization of the chromatin fibers at different compaction levels linked to isochore families increased the DSB yield by 6–10%, and it allowed the most affected part of the genome to be identified. These new results indicate that the genome core is more radiosensitive than the genome desert, with a 3–8% increase in damage depending on the LET. This work highlights the importance of using realistic distributions of chromatin compaction levels to calculate radio-induced damage using Monte Carlo simulation methods.

## 1. Introduction

The ionizing properties of radiation are used in tumor treatment by radiation therapy protocols, commonly with photon beams, as well as in new techniques, such as proton and carbon therapy. Unfortunately, the conformation of the dose to the tumor shape does not completely spare the healthy tissue, and this lack of selectivity leads to the possibility of side-effects within the healthy tissue adjacent to the target volume. These effects, whether stochastic or deterministic, may result from severe alterations in the nuclear DNA molecule, mainly double-strand breaks (DSBs), which are induced by the direct and indirect effects of radiation [1,2]. A better understanding of the induction of these alterations by different radiation qualities is, therefore, crucial in the study of the side-effects of radiation therapy in order to optimize this type of treatment. This better understanding relies on both biological experiments and simulation techniques using Monte Carlo track structure codes [3,4,5,6].

From a biological point of view, several studies have established that the DNA compaction level in the nucleus influences its radiosensitivity, as well as the damage repair pathways [7,8,9,10,11,12,13,14]. The compaction of the DNA molecule is organized in different ways depending on the cell cycle and cell environment. In the G0/G1 phase, the cell’s transcriptional phase, the DNA molecule is organized at the scale of the DNA fiber in two compaction levels: heterochromatin and euchromatin [15,16,17]. Euchromatin is the decondensed form in which the DNA fiber resembles a pearl necklace 11 nm in diameter, the “pearls” being nucleosomes linked by the DNA double helix. Heterochromatin is the condensed form of the fiber, under the compaction action of histone H1, with a solenoid structure of 30 nm in diameter. The biological functions of these forms of compactions are different. Indeed, euchromatin contains the transcribed information, whereas heterochromatin has a structural and gene-silencing function [18].

The contribution of in silico simulation has been crucial in understanding the mechanisms inducing initial DNA damage and its relationship with different forms of DNA compaction. Modeling is generally based on Monte Carlo methods for nanodosimetric calculations [3,4,5,6]. It uses geometrical models of cell nuclei describing the full DNA target that can sometimes include different forms of DNA compaction [19,20]. Indeed, the study on the impact of DNA chromatin compaction on damage topology presented in [20] highlighted that more indirect damage was obtained in euchromatin compared to heterochromatin, thereby possibly increasing radiosensitivity. However, the geometrical model this study used presented a random distribution of euchromatin and heterochromatin in the genome. This leads to the question of the impact that a different and more biologically based type of spatial distribution would have on the simulated DNA damage and its complexity.

Through the proposal of the biological theory of isochores in 1993 [21], Bernardi put forward the fact that the genome contains segments richer in GC than others, to which coding properties are attributed. These segments were first defined as having a size greater than 300 kbp. However, many controversies have emerged about the difficulty of identifying such segments in the genome [22,23,24]. This controversy led to a more flexible definition of the isochores, with respect to segment size [25,26]. Different methods to detect isochores from genomic sequencing have emerged [27]. Among them, the detection method by sliding window [28,29] was identified as the best for detecting isochores of the fixed-size window [27]. Later, Costantini highlighted the fact that the genome consists of a mosaic of isochores with a typical size of 1 Mbp [30]. In our application, we linked the GC content of genome segments to the compaction level of the chromatin fiber that constitutes them, as in the PARTRAC code [3]. This allowed us to access a nonrandom distribution of chromatin fiber compaction levels, through genome sequencing, within our nucleus model.

Therefore, in this study, we developed a new geometrical description of an endothelial cell nucleus in the G0/G1 phase, aiming to refine its DNA geometry according to the isochore theory. This type of cell nucleus was chosen because these developments are part of IRSN’s largest project aiming to better understand the side-effects of hadron therapy in healthy tissue, particularly inflammatory processes in which endothelial cells constitute a key target. Then, we used this new geometrical model as the target in our nanodosimetric simulation chain, which was previously benchmarked against experimental results of early DNA damage induction for different radiation qualities [31,32], in order to calculate the number, location, and complexity of the simulated DSBs and compare the results to previous studies. The simulation chain [31] was based on Geant4-DNA [33,34,35,36] and took into account all the steps leading to early radiation damage, namely, the physical, physicochemical, and chemical stages. The comparison between the results using different geometrical descriptions of the chromatin compaction identified new mechanisms conferring a higher radiosensitivity to regions essentially composed of euchromatin. The results presented in this paper effectively demonstrate that the characteristics of the geometrical model used to describe chromatin compaction distribution have an impact on the simulated results and can explain some of the biological differences between cell types. Therefore, even if this study focuses on one nucleus type (endothelial), these results suggest that the geometric representation of the cell nuclei used in simulation studies must be adapted to each cell type, which can be achieved in our simulation chain thanks to the tools presented in this paper. It is also important to note that the results presented are for initial DNA damage; the simulation results correspond to a picture of all DNA damage produced after the chemical stage and before any repair process has started. Therefore, the calculated results can only be compared with experimental data obtained in vitro, taking into account the experimental biases related to the signaling and repair processes that, in most cases, have already started prior to measurement.

## 2. Results

### 2.1. Impact of the New Geometries on Clustered and Non-Clustered Damages

**Figure 1:**
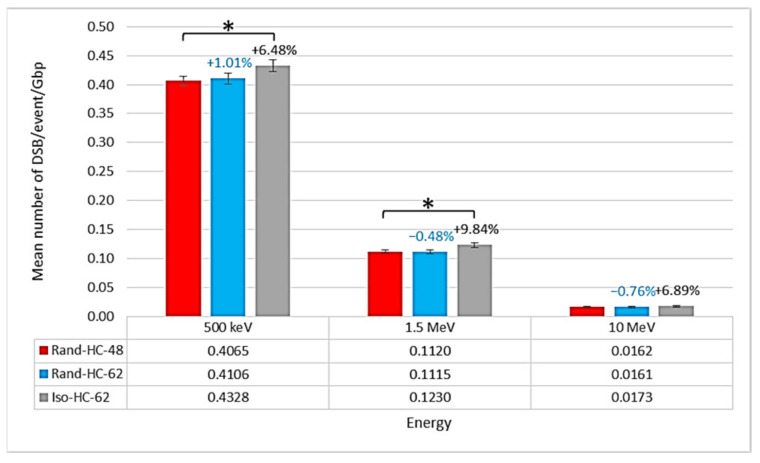
Comparison of the mean number of DSBs/event/Gbp for the three endothelial geometries (Rand-HC-48, Rand-HC-62, and Iso-HC-62). The figures above the bars indicate the relative increase in mean DSBs/event/Gbp number for the Rand-HC-62 and Iso-HC-62 nuclei compared to the Rand-HC-48 nucleus. Asterisks above the bars represent the degree of confidence on the difference between the two related mean values assessed by Student’s *t*-test (* *p* < 0.05).

The results on three types of nuclei are presented here. The new Iso-HC-62 nucleus model was compared to the old Rand-HC-48 model. In order to better identify the parameters that influence the differences between these two models, a third model was included in the study: Rand-HC-62. Then (irradiation setup described in Section 4), the isochore geometry (Iso-HC-62) showed a result of 4.33 × 10^−1^ DSBs/event/Gbp at 500 keV, 1.23 × 10^−1^ DSBs/event/Gbp at 1.5 MeV, and 1.73 × 10^−2^ DSBs/event/Gbp at 10 MeV. For the same irradiation conditions, geometries in which the compaction levels were randomly distributed showed less damage. Indeed, the nuclei Rand-HC-62 and Rand-HC-48 produced 4.11 × 10^−1^ DSBs/event/Gbp and 4.06 × 10^−1^ DSBs/event/Gbp at 500 keV, 1.11 × 10^−1^ DSBs/event and 1.12 × 10^−1^ DSBs/event at 1.5 MeV, and 1.61 × 10^−2^ DSBs/event/Gbp and 1.62 × 10^−2^ DSBs/event at 10 MeV, respectively. As represented in Figure 1, a comparison between the nucleus Rand-HC-48 used in [20] and the new nucleus with an isochore distribution showed a 6–10% increase in the number of DSBs/event/Gbp. However, the number of DSBs/event/Gbp in the nucleus Rand-HC-62 was more or less the same (1%) as that for Rand-HC-48, which only differed for the euchromatin rate. To assess the relevance of the comparisons between the Rand-HC-48 nucleus and the other nuclei, Student’s *t*-tests were performed. These revealed a significant difference, with a *p*-value less than 0.05, with the nucleus Iso-HC-62 at energies 500 keV and 1.5 MeV. At the same time, the tests did not reveal significant deviations with the Rand-HC-62 nucleus. We noticed that, at 10 MeV, the mean number of DSB/event/Gbp increased for the Iso-HC-62 nucleus in the same proportion as at other energies, compared to the Rand-HC-48 nucleus, but the results of the statistical tests did not allow us to qualify this difference as significant.

**Figure 2:**
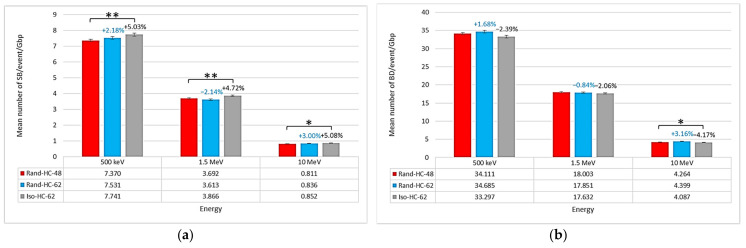
For the three endothelial geometries (Rand-HC-48, Rand-HC-62, and Iso-HC-62) irradiated with protons at different energies, comparison of the mean number of (**a**) strand breaks (SBs)/event/Gbp and (**b**) base damages (BDs)/event/Gbp. For the Rand-HC-62 and Iso-HC-62 nuclei, the figures above the bars indicate the relative increase compared to the Rand-HC-48 nucleus in mean number of (**a**) SBs/event/Gbp and (**b**) BDs/event/Gbp. Asterisks above the bars represent the degree of confidence on the difference between the two related mean values assessed by Student’s *t*-test (* *p* < 0.05, ** *p* < 0.01).

Concerning non-clustered damage, there were several differences. First, Figure 2 shows that the mean number of SBs/event/Gbp was higher for the isochore geometry (7.741 SBs/event/Gbp at 500 keV, 3.866 SBs/event/Gbp at 1.5 MeV, and 0.852 SBs/event/Gbp at 10 MeV) than for the other two geometries. However, the two other geometries produced a similar mean number of SBs/event/Gbp (7.370 SBs/event/Gbp at 500 keV, 3.692 SBs/event/Gbp at 1.5 MeV, and 0.811 SBs/event/Gbp at 10 MeV for the nucleus Rand-HC-48 and 7.531 SBs/event/Gbp at 500 keV, 3.613 SBs/event/Gbp at 1.5 MeV, and 0.836 SBs/event/Gbp at 10 MeV for the nucleus Rand-HC-62). This comparison was supported by the results of Student’s *t*-test between the Rand-HC-48 nucleus and the other two nuclei. Indeed, the test revealed that the differences between the Iso-HC-62 and Rand-HC-48 nuclei were significant, with confidence levels lower than 0.01 for the energies of 500 keV and 1.5 MeV, respectively, and lower than 0.05 for 10 MeV. On the other hand, Student’s *t*-test did not reveal significant differences between the Rand-HC-48 and Rand-HC-62 nuclei. Concerning the mean number of BDs/event/Gbp, the trend was reversed since there were more BDs/event/Gbp in the random geometries than in the isochore geometry. Nevertheless, we could only observe a trend to be qualified by the fact that the Student’s *t*-tests on the 500 keV and 1.5 MeV energies did not produce *p*-values below the 0.05 criterion (0.07 for 500 keV and 0.12 for 1.5 MeV). This was due to the small difference between the mean number of BDs/event/Gbp of the Rand-HC-48 and Iso-HC-62 nuclei and the uncertainty associated with these values.

**Figure 3:**
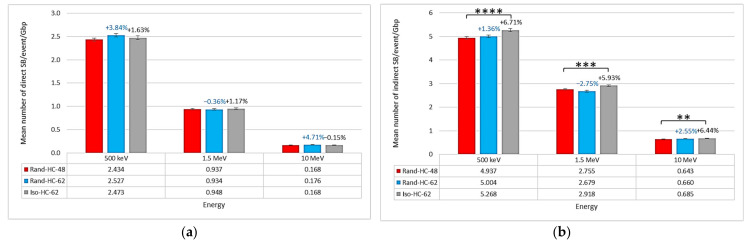
For the three endothelial geometries (Rand-HC-48, Rand-HC-62, and Iso-HC-62) irradiated with protons at different energies, comparison of the mean number of (**a**) direct SBs/event/Gbp and (**b**) indirect SBs/event/Gbp. For the Rand-HC-62 and Iso-HC-62 nuclei, the figures above the bars indicate the relative increase compared to the Rand-HC-48 nucleus in mean number of (**a**) direct SBs/event/Gbp and (**b**) indirect SBs/event/Gbp. Asterisks above the bars represent the degree of confidence on the difference between the two related mean values assessed by Student’s *t*-test (** *p* < 0.01, *** *p* < 0.001, **** *p* < 0.0001).

Regarding the type of damage in the different geometries, Figure 3 shows that the isochore geometry presented a higher mean number of indirect (produced during the chemical stage) SBs/event/Gbp (5.268 SBs/event/Gbp at 500 keV, 2.918 SBs/event/Gbp at 1.5 MeV, and 0.685 SBs/event/Gbp at 10 MeV) than the other two geometries. On the other hand, the mean number of direct SBs/event/Gbp was about the same for the three geometries. These observations were supported by Student’s *t*-tests that indicated a significant difference, with a high level of confidence, in the mean number of indirect SBs/event/Gbp between the Rand-HC-48 nucleus and the Iso-HC-62 nucleus. At the same time, the tests did not reveal significant differences between the Rand-HC-48 and Rand-HC-62 nuclei in the mean number of direct and indirect SBs/event/Gbp, nor between the Rand-HC-48 and Iso-HC-62 nuclei in the mean number of direct SBs/event/Gbp.

### 2.2. Location of Damages in the Isochore Nucleus

**Figure 4:**
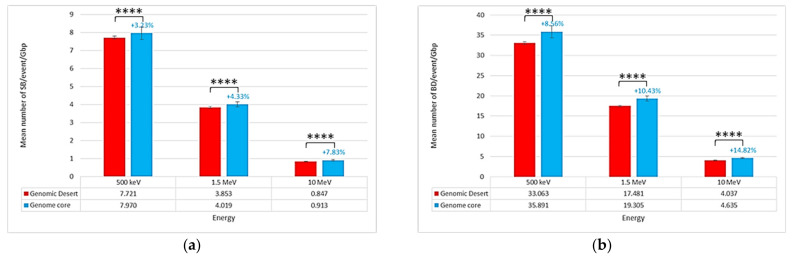
Location of the simulated damage produced by proton (**a**) SBs/event/Gbp and (**b**) BDs/event/Gbp. For the genome core, the figures above the bars indicate the relative increase compared to the genomic desert in the mean number of (**a**) SBs/event/Gbp and (**b**) BDs/event/Gbp. Asterisks above the bars represent the degree of confidence on the difference between the two related mean values assessed by the Mann–Whitney U-test (**** *p* < 0.0001).

Figure 4 shows the strand break locations in different regions of the isochore nucleus: the genomic desert (corresponding to families L1, L2, and H1) and the genome core (H2 and H3 families). Interestingly, the SB yield was not uniformly distributed within these regions, as the total number of SBs/event/Gbp was higher in the genome core (7.970 SBs/event/Gbp at 500 keV, 4.019 SBs/event/Gbp at 1.5 MeV, and 0.913 SBs/event/Gbp at 10 MeV) than in the genomic desert (7.721 SBs/event/Gbp at 500 keV, 3.853 SBs/event/Gbp at 1.5 MeV, and 0.847 SBs/event/Gbp at 10 MeV). This was also the case for the total number of BDs/event/Gbp (35.891 BDs/event/Gbp at 500 keV, 19.305 BDs/event/Gbp at 1.5 MeV, and 4.635 BDs/event/Gbp at 10 MeV in the genome core vs. 33.063 BDs/event/Gbp at 500 keV, 17.481 BDs/event/Gbp at 1.5 MeV, and 4.037 BDs/event/Gbp at 10 MeV in the genomic desert). Mann–Whitney U-tests applied to these data revealed significant differences in the results between the genome core and the genomic desert. Here, a nonparametric test was preferred to Student’s *t*-test because the data distributions were not Gaussian.

### 2.3. Single-Voxel Study

**Figure 5:**
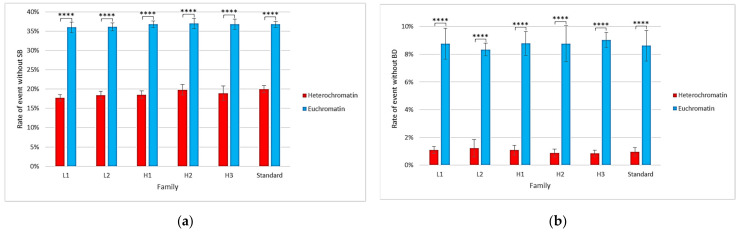
Rate of particle tracks for voxels of different families without induction of (**a**) SB and (**b**) BD. Error bars were obtained with five simulations. Asterisks above the bars represent the degree of confidence on the difference between the two related mean values assessed by Student’s *t*-test (**** *p* < 0.0001).

Single-voxel simulations provide more information about voxel-specific differences. Figure 5 shows that the proportion of events (proton tracks) not producing damage (SB or BD) was different in heterochromatin and euchromatin voxels. Indeed, the probability of not having an SB for a track crossing a euchromatin voxel was approximately 37%, while it was only 18% for a heterochromatin voxel. For BD, 9% of events were without any damage in the euchromatin voxel vs. 1% in the heterochromatin voxel. Student’s *t*-tests applied to these data revealed significant differences in the results on heterochromatin and euchromatin voxels.

**Figure 6:**
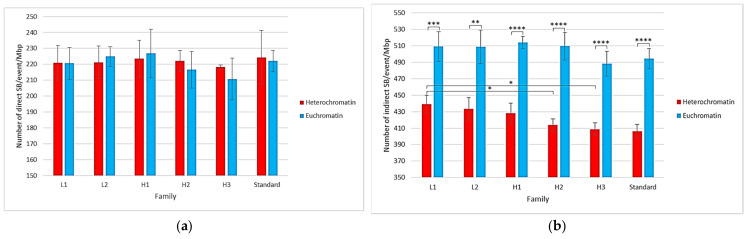
Comparison, for each type of voxel family, of the mean number and yield of (**a**) direct SBs/event/Mbp and (**b**) indirect SBs/event/Mbp. Error bars were obtained with five simulations. Asterisks above the bars represent the degree of confidence on the difference between the two related mean values assessed by Student’s *t*-test (* *p* < 0.05, ** *p* < 0.01, *** *p* < 0.001, **** *p* < 0.0001).

Figure 6 focuses on the type of SB in the heterochromatin and euchromatin voxels of different families. It shows that the mean number of direct SBs/event/Mbp was the same for voxels of all types (around 220 SBs/event/Mbp), regardless of the family. Regarding the mean number of indirect SBs/event/Mbp, it was higher in euchromatin voxels (approximately 500 SBs/event/Mbp) than in heterochromatin voxels (approximately 410 SBs/event/Mbp). This comparison was credited with a high level of confidence by the Student’s *t*-tests performed on these data. Similarly, there was a slight trend suggesting that there would be more damage in voxels with less GC-concentrated families, as the distribution of direct/indirect damage for heterochromatin voxels was around 34%/66%, while it was 30%/70% for euchromatin. Student’s *t*-tests revealed, for example, a significant difference, with a *p*-value lower than 0.05, between the mean number of indirect SBs/event/Gbp in the heterochromatin voxels of the L1 family and those of the H2 and H3 families.

## 3. Discussion

As discussed in the introduction, the DSB is one of the most critical types of radiation-induced damage. Therefore, it is first necessary to verify the consistency of the simulated DSB yields obtained with the isochore nucleus’s new geometric model with respect to available experimental data, even if the direct comparison of simulation results with experimental data is made difficult by the experimental limitations in the detection of DSBs [37]. Unfortunately, there are few experimental measurements on endothelial cell nuclei (our project cell model), and the available literature on proton-induced DSB-measured data for other cell types does not allow the influence of the geometrical isochore model in the simulated results to be verified with respect to those previously obtained, as mentioned in the introduction. Indeed, the differences in the various chromatin compaction models shown in the results section are just a few percentage points, while differences in the experimental DSB data for different cell types are much higher [38]. Nevertheless, our simulation chain was fully benchmarked in a previous study [39]. It showed that the mean number of DSBs/event/Gbp calculated with our simulation chain coupled to different cell nuclei models, including the Rand-HC-48 nucleus, was in acceptable agreement with the experimental data available in the literature. With the new distribution of chromatin compaction levels in the Iso-HC-62 nucleus, the mean number of DSBs/event/Gbp increased from 6% to 10% over the three simulated proton energies compared to the Rand-HC-48 nucleus. This reasonable increase in the number of DSBs/event/Gbp is, therefore, acceptable according to the literature.

The small difference in the number of DSBs/event/Gbp between the two random nuclei (approximately 1% difference as shown in Figure 1) suggests that the difference between the Rand-HC-48 nucleus and the Iso-HC-62 nucleus was not due to the overall heterochromatin rate. If this was the case, the number of DSBs/event/Gbp obtained with the Rand-HC-62 nucleus would not have been the same as that obtained with the Rand-HC-48 nucleus. However, it should be noted that the number of DSBs/event/Gbp directly depends on the classification process applied to the raw damage topology, as explained in Section 4. Therefore, it is appropriate to consolidate these observations with a direct analysis of non-clustered damage results (number of SBs/event/Gbp). Referring to Figure 2, the previous observation is supported by the fact that non-clustered damage followed the same trend. This confirms that it was not the modification in the overall heterochromatin rate that induced a difference in the number of DSBs/event/Gbp, but rather its distribution using the isochore model.

The results presented in Figure 3b show that this difference was induced by the number of indirect SBs/event/Gbp. Indeed, the number of direct SBs/event/Gbp was roughly equal for each of the three nuclei (see Figure 3a), while the number of indirect SBs/event/Gbp was higher in the isochore nucleus than in the other nuclei. A deeper analysis of the damage location in the isochore nucleus (see Figure 4) revealed that there were more SBs/event/Gbp in the genome core than in the genome desert. As a reminder, the genome core is made up of segments of the H2 and H3 families and, therefore, presents a high level of euchromatin (Table 1). The hypothesis resulting from these two observations is that the creation of segments consisting entirely or predominantly of euchromatin induces an increase in overall damage. Indeed, euchromatin voxels are more sensitive to chemically produced SB than heterochromatin voxels, as previously published elsewhere [20] and confirmed by the analysis of the voxel response in Figure 6. Nevertheless, this contradicts the fact that no difference was observed between the two random nuclei for which the heterochromatin rates were quite different (48% vs. 62%). To clarify this contradiction, one can argue that euchromatin voxels show less frequent damage than heterochromatin voxels when they are crossed by an event, as shown in Figure 5. This implies that a geometric bias can occur in our models due to the spatial concentration of the voxels in the fiber governed by the filling algorithm. Indeed, a higher level of heterochromatin on a 1 Mbp segment of the genome results in a lower spatial concentration of voxels. Therefore, the linear correlation between the heterochromatin rate and the amount of damage in our model is not obvious due to the difference in the spatial density of voxels. On the other hand, the genome core is only a small part of the genome (approximately 11% in our model); therefore, the higher damage observed in this region only slightly influenced the overall number of SBs/event/Mbp. Nevertheless, this increase should have a major biological impact since it constitutes a large part of the coding genome.

The increase in damage in the isochore nucleus compared to the random nuclei is also explained by the GC rate in voxels influencing the BD/SB distribution since reaction rates in the chemical stage may differ significantly for some bases (Table 2). It is important to note, when comparing Figure 2a and Figure 4a, that the number of SBs/event/Gbp in the genomic desert of the isochore nucleus remained higher than the number of SBs/event/Gbp in the two random nuclei when it was predominantly composed of heterochromatin. Referring to Table 2, guanine has a stronger reaction rate with hydroxyl radicals, which is responsible for indirect damage to the DNA backbone. This suggests that, by decreasing GC levels and, therefore, guanine, in the voxels that make up the genomic desert, the bases would consume fewer hydroxyl radicals, leading to an increase in backbone damage and, thus, an increase in the number of SBs/event/Gbp. This assumption is also supported by the comparison of Figure 2b and Figure 4b, which highlights higher base damage in the genome core of the isochore nucleus than in the random nuclei and less damage in the genomic desert. Lastly, a trend can be noted in Figure 6, which suggests that the number of indirect SBs/event/Mbp in heterochromatin voxels would increase with a lower GC rate in the voxel in question (i.e., depending on the voxel family). The phenomenon described here is, therefore, intimately linked to the value assigned to the reaction rate between guanine and the hydroxyl radical. It must be noted that this reaction rate has never been directly measured in neutral conditions, unlike the reaction rates of other bases with this radical. If this reaction rate was revised upward, this effect would be even more significant. If it was revised downward, this effect would be erased. It appears, however, that guanine remains the most reactive molecular species of the four bases [40,41,42,43,44,45].

To summarize, there are two mechanisms directly linked to the isochore model that can explain the increase in simulated damage with this model compared to other nuclei models which do not consider chromatin compaction localization in the genome. The first mechanism is that the creation of segments consisting entirely or predominantly of euchromatin induces an increase in the number of SBs on these segments due to the higher induction of indirect effects. The second mechanism is that the modification of the GC rate in the new isochore voxel models influences BD/SB distribution, which induces an increase in the number of global SBs and, a fortiori, in the genomic desert, which has a lower GC level. Due to the composition of the isochore genome (89% genomic desert vs. 11% genome core), it is easy to infer that the impact of second mechanism on the increase in global SB number is more significant than the first. Nevertheless, the first mechanism is still important because it occurs in the coding part of the genome, rendering it much more radiosensitive than the genomic desert (see Figure 4).

This study shows that the difference in damage induction between the old geometrical models and the isochore model is small (approximately 10%) but significant. Moreover, this level of precision in chromatin distribution also allows significance to be assigned to the calculated damage for the first time due to its localization. This study, therefore, highlights the importance of realistically modeling the distribution of chromatin compaction levels at the cell nucleus scale. This makes the results obtained with this new nucleus model an interesting database for repair models that consider the level of compaction of the chromatin fiber in the choice of repair pathway. It is also planned to implement repair models in the Geant4-DNA simulation chain in order to predict the outcome of damage processing in terms of cell death or chromosome aberrations, for example. The direct experimental validation of the results presented in this paper remains difficult because no experimental data have been acquired at a sufficiently fine level of detail to evaluate the greater radiosensitivity of the genome core highlighted in our results. The validation of such an observation should be based on the separate induction of damage in the heterochromatin or euchromatin areas. As such, an experimental project is under development at IRSN for targeted irradiation of heterochromatin and euchromatin areas of endothelial cells using a microbeam.

## 4. Materials and Methods

### 4.1. Geometry Modeling Tool

**Figure 7:**
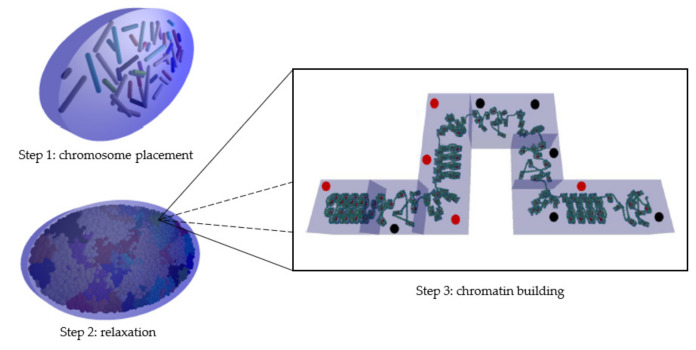
Illustration of the three steps needed to build the endothelial nuclear geometry. In step three, heterochromatin voxels are marked with red dots and euchromatin voxels are marked with black dots.

DnaFabric software [6] was used to build the complete nuclear geometry including the continuous chromatin fiber down to the base pair (bp) precision level of a human endothelial cell in the G0/G1 phase. To build this fiber, the three steps presented in Figure 7 are required once the nucleus shape and dimensions are defined:

- The first step consists of placing the 46 chromosomes in a condensed form (not biological; for more information, please see [6]) at their preferred position within the nucleus.
- The second step expands these condensed chromosomes into connected spherical domains of 1 Mbp to occupy maximum space in the nucleus [46].
- The third step consists of building the chromatin fiber placing one by one the cubic voxels (40 nm sides) in the spherical domains and ensuring that they are continuously connected with each other.

Five voxels of different orientations (straight, up, down, left, and right) were predefined in the two well-known compaction levels: euchromatin and heterochromatin. The guanine/cytosine (GC) rate of the standard voxel was 50, the global composition of the cell nuclei generated using DnaFabric software comprised 48% heterochromatin voxels and 52% euchromatin voxels, and the voxels were randomly distributed throughout the genome, as described in our previous work [20]. In the construction of the chromatin fiber, the space occupation in each domain was maximized by determining the voxel direction using the different algorithms described in [6].

### 4.2. New Model Geometry Based on the Isochore Theory

Previously presented in the PARTRAC code [19], the isochore model was used in this work to render our geometries more representative of chromatin fiber compaction along the genome. This biological model was proposed by Costantini et al. in 2006 [29], and then confirmed in 2017 [30] to describe chromatin fiber compaction in the G0/G1 phase, in particular. This model demonstrates that the different genome segments could be classified into five families according to the quantity of GC bases each segment contained. Moreover, the chromatin fiber’s compaction level in the segment would be directly related to the family in which the segment is classified.

Generating this new geometry with DnaFabric software requires a complete isochore mapping of the genome; the tool proposed by Jan Pačes [28] was modified and used for this. A code in PERL language provides a graphical overview of the location of the segments belonging to each of the five isochore families within each chromosome. In this work, the most recent version of the human reference sequence (GRCh38), established in December 2013 [47], was used. This genome was in the form of FASTA files (one file per chromosome pair, one file for the X chromosome, and another for the Y chromosome), a format used to store biological nucleic or protein sequences. These sequences are represented by a sequence of alphanumeric characters encoding nucleic acids or amino acids according to the International Union of Pure and Applied Chemistry nomenclature [48].

To analyze the base sequence of a chromosome, a 1 Mbp sliding window moving with a constant step of 1 Mbp was chosen. The size and step of this window were chosen on the basis of the chromatin fiber’s organization in 1 Mbp spherical domains in our geometrical model. Indeed, to avoid creating hybrid domains (i.e., containing segments of different families) during domain filling, a family was assigned to each domain of our model. On each application of the window, the GC rate was calculated on the genome segment studied. Thus, once the entire genome was analyzed, the GC concentration mapping was obtained. Then, by analogy, as described in Table 1, each segment was classified into one of the five isochore families with respect to its GC concentration, and a heterochromatin rate was associated with it. These heterochromatin rates were fixed in order to respect the gradation in the compaction level of each of the families, with the L1 family being assimilated to pure heterochromatin, and the H3 family being assimilated to pure euchromatin.

Each domain was filled respecting the heterochromatin/euchromatin voxel rate assigned by its isochore family. However, to be consistent with the isochore theory, new voxels were developed. Keeping the geometry of standard voxels (straight, up, down, left, and right for heterochromatin and euchromatin), the GC rate of these voxels was adjusted according to their isochore family. Thus, L1 voxels had a GC rate of 35%, L2 had 40%, H1 had 45%, H2 had 50%, and H3 had 55%. Overall, this new cell nucleus geometry had a global rate of 62% of heterochromatin voxels and 38% of euchromatin voxels. This rate preserves the agreement with the previous nucleus version and the heterochromatin and euchromatin rates measured [20].

### 4.3. Damage Calculation

Starting from the simulation chain developed in our group and presented in [20,31], an updated version of the simulation chain based on the Geant4-DNA version 10.6 was used to simulate the quantity and the topology of early radiation-induced damage [31]. The direct interactions of radiation with the DNA molecule (physical step) were modeled, as well as the creation of free radicals in the medium near the DNA (physicochemical step) and the diffusion and reactions of these radicals between them and with DNA molecules (chemical step).

For the simulation of the physical step, the Geant4-DNA physics constructor option 2 (default) was used. In our simulation, the interaction cross-sections of the target, including base pairs, DNA backbone, and the hydration shell, were assimilated to liquid water. For the simulation of the chemical step, the constructor used was also the default of Geant4-DNA, making use of the step-by-step approach [49]. The simulation time of the chemical step was set to 5 ns in order to match the average range of the hydroxyl radical in the biological environment in the literature [50,51] and maintain the simulation parameters for comparison with previous results. The reaction rates between radicals and DNA constituents are listed in Table 2, taken from Buxton [52]. It should be noted that the reaction rate of guanine with the hydroxyl radical was derived from a measurement carried out under non-neutral conditions (pH = 10) [53], since guanine base alone cannot be dissolved in water. Indeed, to our knowledge, there is no measurement in the literature of the rate of reaction of guanine with the hydroxyl radical under neutral conditions. Nevertheless, several studies suggest that guanine is the most sensitive base to oxidation [40,41,42,43,44], especially to the oxidizing agent OH^•^ [45]. This trend was also observed experimentally by electrophoresis measurements [54]. In view of these elements, we decided to keep the reaction rate of 9.20 × 10^9^ M^−1^·s^−1^ for the reaction between guanine and the hydroxyl radical, as in the PARTRAC code [55] or former results using Geant4-DNA-based modeling [5,6,56]. It is nevertheless important to keep in mind that this value does not result from a direct measurement. Furthermore, regarding the damage production rate, in this simulation, we considered that a base damage was produced during the chemical stage every time a reaction between a hydroxyl radical and a base happened. For the strand break productions during the physical or the chemical stages, the same parameters as those used in our previous work [20,31] were used. It can be noted that the hydroxyl radical has a major role in the induction of indirect damage to the bases and the DNA backbone, as described in the literature [57]. It should also be noted that the high rate constants are mainly related to the action of hydroxyl radicals on the DNA bases rather than on the sugar. This is the reason why the rate of production of strand breaks in DNA is low, as recalled by von Sonntag [45].

Once the locations of direct and indirect strand breaks (SBs), as well as base damages (BDs), in the genome were known, the final damage classification was performed for double-strand breaks (DSBs), single-strand breaks (SSBs), and base damages (BDs). For this classification, in a first step, SBs with an interval of 10 bp or less were clustered. A cluster was considered a DSB if at least two of the SBs in it were located on two different strands. If all the SBs in the cluster were located on the same strand, then the cluster was classified as an SSB. The number of SBs in the cluster determines the complexity of the DSB or SSB. Once this first clustering was performed, each cluster was “enriched” with the BD inside it or with distance of less than 5 bp.

### 4.4. Configurations, Cell Nucleus Geometries, and Simulation Parameters

Two types of simulations were performed: complete cell nucleus and isolated voxels. For the full nuclei simulations, the damage in three different geometries were compared: the nucleus used so far (random distribution, 48% heterochromatin/52% euchromatin, abbreviated Rand-HC-48), the new isochore nucleus (isochore distribution, 62% heterochromatin/38% euchromatin, abbreviated Iso-HC-62), and a specially designed nucleus with a random distribution of the voxels respecting the global heterochromatin and euchromatin rates of the isochore nucleus model (random distribution, 62% heterochromatin/38% euchromatin, abbreviated Rand-HC-62). Irradiation was composed of charged particles emitted vertically toward the nucleus from the surface of an ellipse of radii 4 µm and 8 µm, at a distance of 5 nm from the upper surface of the nucleus (see Figure 7). Simulations of 1000 protons of 500 keV (LET: 43.25 keV/µm), 1500 protons of 1.5 MeV (LET: 19.51 keV/µm), and 2000 protons of 10 MeV (LET: 4.29 keV/µm) were performed on each of the two random endothelial cell nucleus geometries. Simulations of 1500 protons of 500 keV, 2500 protons of 1.5 MeV, and 3000 protons of 10 MeV were performed on the isochore endothelial cell nucleus geometry. The number of protons for each energy and each nucleus was chosen in order to have enough statistical power and to be sure to irradiate the total cell nucleus volume. Indeed, a higher LET leads to higher ionization density, more damage, and consequently, lower uncertainty.

**Figure 8:**
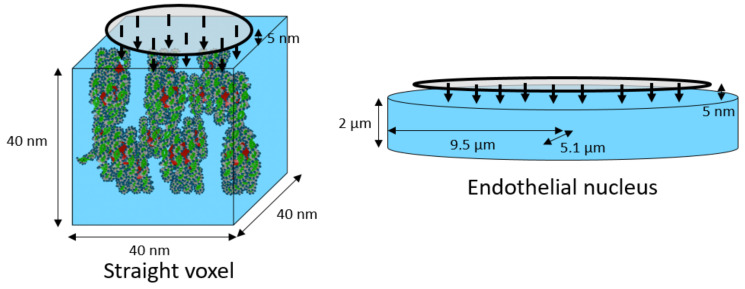
Geometry of simulated irradiations for the irradiation of a single voxel (**left**) and for the irradiation of a HUVEC cell nucleus model (**right**) [20] containing the DNA geometry (not shown here).

Moreover, simulations were performed considering simple geometries containing only one voxel. Then, the effect of changing the GC rate in voxels, as well as the effect of fiber condensation for two voxels of the same orientation, was studied. For this purpose, five simulations of 1000 protons of 500 keV were performed on each of the six types (L1, L2, H1, H2, H3, and standard) of straight euchromatin and heterochromatin voxels. Heterochromatic voxels of each family contained 18 nucleosomes and 3594 bp, while euchromatic voxels contained 10 nucleosomes and 2011 bp. Particles were emitted vertically toward the voxel from the surface of a circle with a 20 nm radius, 5 nm from the voxel’s upper surface (see Figure 8).

## 5. Conclusions

In this study, we assessed the influence of a more realistic geometry of chromatin compaction in a cell nucleus in simulated early DNA damage obtained with a simulation chain based on Geant4-DNA. The results obtained with this new isochore geometric model revealed the number of calculated DSBs/event/Gbp in agreement with experimental values from the literature that were previously found with a former random chromatin distribution while providing a biological basis for the arrangement of chromatin along the genome. This new model also allowed us to access a nonrandom distribution of base pairs directly related to fiber compaction and the location of critical damage for cell fate by introducing the notion of the genome core and genomic desert. Effectively, the genome core, the coding part of the genome, presents a surplus of damage compared to the genomic desert as a result of its essentially euchromatic constitution. This phenomenon is due to the particular radiosensitivity of the euchromatin to indirect damage.

## Figures and Tables

**Table 1:** Properties of the different isochore families in terms of GC rate, as well as the associated heterochromatin/euchromatin composition.

Family	GC Rate	Heterochromatin Content
L_1_	<37.5%	100%
L_2_	37.5–42.5%	70%
H_1_	42.5–47.5%	50%
H_2_	47.5–52.5%	20%
H_3_	>52.5%	0%

**Table 2:** List of reactions considered in the simulation and their associated reaction rates.

Reactions	Reaction Rates (10^9^ M^−1^·s^−1^)
2-Deoxyribose + OH^•^	1.80
Adenine + OH^•^	6.10
Guanine + OH^•^	9.20
Thymine + OH^•^	6.40
Cytosine + OH^•^	6.10
2-Deoxyribose + e^−^_aq_	0.01
Adenine + e^−^_aq_	9.00
Guanine + e^−^_aq_	14.00
Thymine + e^−^_aq_	18.00
Cytosine + e^−^_aq_	13.00
2-Deoxyribose + H^•^	0.029
Adenine + H^•^	0.10
Thymine + H^•^	0.57
Cytosine + H^•^	0.092

## Data Availability

Not applicable.
